# Thoughtful surgical practice for therapeutic self: A randomized control trial

**DOI:** 10.12669/pjms.36.7.3038

**Published:** 2020

**Authors:** Tausief Fatima, Rehan Ahmed Khan, Faryal Azhar, Usman Mahboob

**Affiliations:** 1 Tausief Fatima, MBBS, FCPS, MHPE, MRCS. Associate Professor of Surgery, Islamic International Medical College, Riphah International University, Rawalpindi - Pakistan; 2 Rehan Ahmed Khan, MBBS(Pak), FCPS (Pak), FRCS (Ire), JM-HPE (Ned), MSc HPE (UK), PhD (Scholar) University of Maastricht (Ned). Professor of Surgery, Islamic International Medical College, Riphah International University, Rawalpindi, Pakistan; 3 Faryal Azhar, MBBS. MRCS. FCPS. Assistant Professor of Surgery, Rawalpindi Medical College Rawalpindi, Pakistan.; 4 Usman Mahboob, Director, Institute of Health Professions Education & Research, Khyber Medical University (KMU), Peshawar, Pakistan

**Keywords:** Reflective practice, Clinical skill, Surgical training activities, Clinical skill competencies

## Abstract

**Objectives:**

To determine the role of structured reflection in teaching basic surgical skills in undergraduate students.

**Methods:**

A randomized control trial was done in two medical colleges of Punjab, from January to December 2017 in which participants were divided into two groups through stratified random sampling. Structured reflection was done by interventional group after training. Post-test was taken to assess their surgical skills. Independent t-test compared the mean of two groups. One-way ANOVA was calculated to measure the difference within the different sub-categories of experimental group.

**Results:**

Out of 140 students that participated in the study, 138 students stayed till the end (retention rate 98.5%, attrition rate 1.5%). Independent t-test (p-value = 0.01) showed statistically significant difference in both control and interventional groups. One-way ANOVA with robust test of equality of Means showed a positive relationship of reflective capacity and acquisition of surgical skills.

**Conclusion:**

The novices who demonstrates better reflective capacity exhibit better acquisition of surgical skills.

## INTRODUCTION

Reflective practices guide the students to flourish. Reflection has many descriptions, but all these come to its origin which means ‘to turn back’.^1^ In the teaching background, reflection can be considered as a sequence of thoughts which are turned back for learning purposes. Reflection can be learnt, taught and assessed to the students in many situations. ‘The Reflection Evaluation for Learner Enhanced Competencies Toll’ (REFLECT) is used to assess reflections and helps to decide student’s overall writing spectrum. This categorises the capacity of reflective writing into critical reflectors, reflectors, thoughtful and habitual.

Medical students can use reflection to learn different skills, attitudes and behaviours needed for clinical practice.^2^ Many studies reviewing skill attainment^3^-^5^ shows possible linkage between reflection and skill attainment. Reflection on any experience will help in identification of the learning needs. Application of newly acquired knowledge comes in next phase. This can be a cyclic process and learning is enhanced in each cycle.^6^

Problems regarding teaching surgical skills include patient safety in busy clinical schedules. Despite the fact that current literature^7^,^8^ supports the importance of reflection, few studies^9^ have examined the relationship of skill attainment and quality of reflection. The rationale of the study is exploring the relationship between knowledge, reflective capacity and skill attainment to provide another viewpoint on usage of reflection. This can help in teaching the clinical skill after exploring the relationship of reflective capacity to the basic surgical skill attainment.

## METHODS

This was a randomized control trial to determine the probable cause and effect between attaining the surgical skills and reflective practice and to see the relationship of reflective power with the acquisition of surgical skill.

This study was conducted in Islamic International Medical College (IIMC) and Rawalpindi Medical College (RMC) from January to December 2017 after IBR approval (Ref # Ripah/GHMS/ERC/17/0178, Dated: January 17, 2017) from both institutes. A total of 430 students of 4^th^ year MBBS, in which 99 students (69=females, 30=males) were from IIMC and 331 students (228=females, 103=boys) were from RMC were the target sample of our study.

After informed consent the sample population calculated through power analysis formula^10^ was found to be 140 and selected through stratified probability sampling to balance the male and female ratio. A selection test to assess their current knowledge of basic Surgical Skill was conducted. The students who score an average or high in the test was excluded, as only novices were included in the study (Table-I).

**Table-I:** Mean score of post interventional test in control and experimental group. (Mean score of surgical skill which is very low in selection test and highest in post intervention test.).

Test	Group	Mean	Standard deviation	T-test for equality of means (p-value)
Selection test		1.63	4.28	
Pretest		18.44	3.63814	
Post intervention score	Control	17.05	4.08687	.01
	Experimental	22.16	4.17421	

After selection test the students were taught the basic surgical skills in groups of 20 pupils (seven groups), then the pre-test was taken, and mean score and standard deviation was calculated (Table-I). Minimum score was seven and maximum was 26 out of 27. Maximum number of students lies between the score of 15-22 which is the range for the middle and high scorers. Only two students were still at the low score, 66 were the middle scorer and 69 were the high scorer.

The result of pre-test is used to divide students into three categories which were: High scorer (19-28 marks on pre-test), Middle scorer (10-18 marks on pre-test), and Low scorer (0-9 marks on pre-test). Students from each category was equally divided into control and experimental group.

After the pre-test and random assignment allocation, the students of the interventional group were taught how to reflect. They were given the proforma comprising nine structured questions on reflection regarding learning the basic surgical skills. This written reflection was assessed and scored according to REFLECT rubric. Overall ratings were scored from 1-4 which is as follows: (1) habitual (no reflection present), (2) thoughtful (some reflection present), (3) reflective (continuously written in reflective tone), and (4) critical reflective (evidence of reflection with change in perspective).

The surgical skill scores were measured both in pre-test and post-test using the continuous scale through OSAT (objective structured assessment of technical skills) and were measured by two examiners and the mean of surgical skill scores were calculated. Results of post intervention test are shown in Table-I.

The data was entered and analysed using SPSS version 22. The unpaired t-test was analysed to see the group difference and One-way ANOVA find the difference between the different sub-groups.

## RESULTS

**Fig.1:**
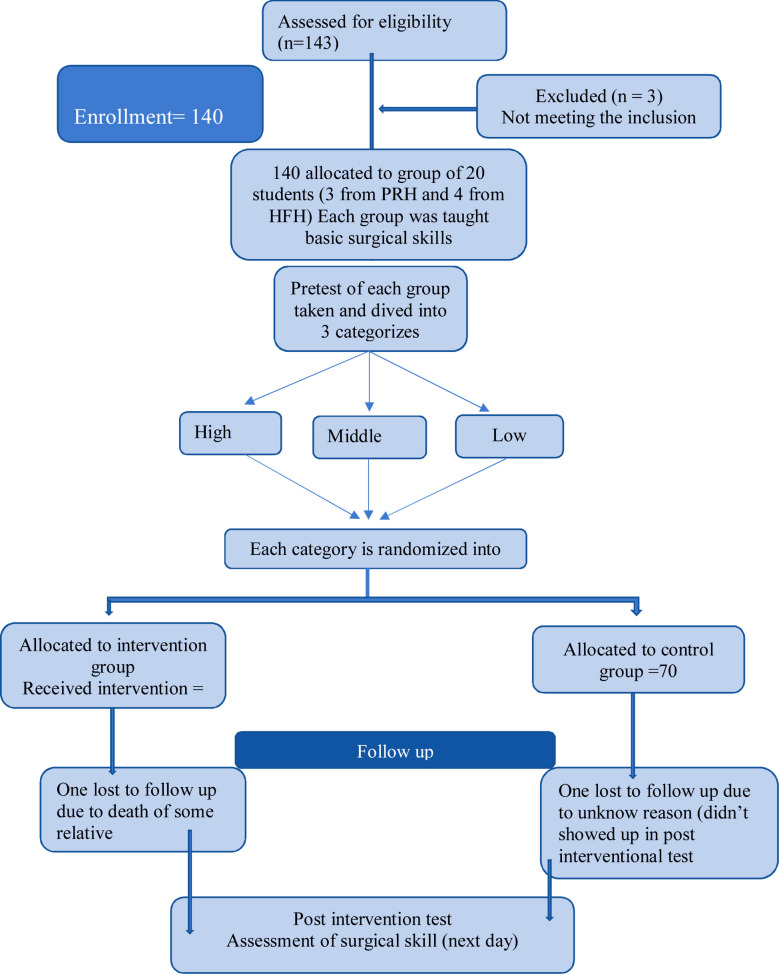
Random allocation of students in control and experimental groups. (Random assignment) randomized using random number table into control and experimental group.

Among 138 students that participated, 92(66.7%) were females and 46(33.3%) were males. (Fig.1).Results of post intervention test are shown in Table-I. The smallest value was 18. After post-test 86 (63%) are high scorer, 46 (33%) are middle scorer and five (4%) are the low scorer.

The reflective capacity was assessed by two examiners. There were 16 (11.6%) habitual, seven (5.1%) thoughtful, 27 (19.6%) reflective and 19 (13.8%) critically reflective students in experimental group. The unpaired t-test compared the means of two groups (Table-I). There was an increase in mean score of surgical skill acquisition in the experimental group. The p-value was 0.01, i.e. a statistically significant difference was found between post interventional test mean score of control and experimental group.

**Fig.2:**
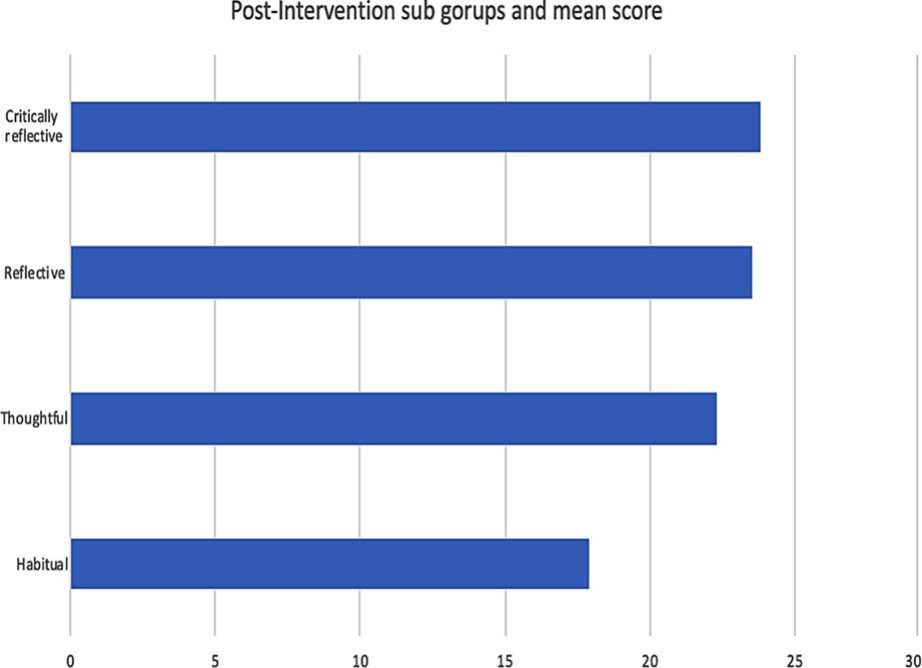
Shows that there is marked difference between the mean score of habitual reflective capacity and other three reflective capacities. But there is not any statistically significant difference between mean score of thoughtful, reflective and critically reflective capacities.

ANOVA test was also conducted to determine if there is a significant difference in mean surgical skill score and the four groups of reflective capacities. It showed significant difference between habitual reflective capacity and reflective and critically reflective capacity (p-value .004 and .002) but there was no statistically significant difference between habitual and thoughtful reflective capacity (p-value .07) (Fig.2). This proved that strength of reflective practice is directly proportional to acquiring surgical skill. Contamination was avoided by sending control group to home while experimental group stayed for reflection and post intervention test was taken next day in different rooms.

## DISCUSSION

There is lot of debate on teaching surgical skills with regards to patient safety busy clinical duties and lack of structured skill teaching in curriculum.^11^,^12^ This research evaluated the impact of structured reflection on surgical skill acquisition. Medical student (novices) learning new skill by reflecting showed improved performance. These results establish the positive relationship of structured reflection to learning while acquiring the basic surgical skill. Though the performance of control group was as good as the experimental group immediately after learning. However, the control group made more mistakes while performing surgical skills in post intervention test as they didn’t practice reflection which can help to incorporate new knowledge in previous experience.

There was a marked difference in learning, between the students who exhibited the habitual reflective capacity and the students who exhibited thoughtful, reflective and critically reflective capacities. The students who exhibited the habitual reflective capacity were not statistically different from the control group in terms of retention of learning basic surgical skills. Students who exhibited the other three categories of reflective practice were not significantly different from each other in terms of the scores in post-intervention test. The findings of this research suggest that learners can acquire surgical skill when they are involved in structured reflection.

The explanation for this change in learning ability is, that reflection aids in the reorganization of challenges faced in current situation. Knowing about self, situation and challenges has a higher impact on learning.^13^ ‘Therapeutic self’, identifies one’s standards and values which are considered as specialised stances and skills.^14^ Knowing *‘self’* is also mandatory to progress the vital *self-efficacy* part that is obligatory to develop a *self-regulated* lifetime student.^14^ Reflection helps in understanding the situation, one will not only restore practice, but these understandings can also change future responses to circumstances.

Learning is enhanced through experience. This cycle of learning can be acquired through experience and reflection.^13^ Imparting skills in students outside the operation theatre is important, as direct practice on the patient is not ethical and safe.^15^ Teaching new skills require explicit training and practice.^16^ Reflection can develop a tendency for learners to process emotions. The process of reflection brings emotional reaction to students consciousness^17^ and helps them for future practice.

Thompson^18^ talks about worth of commitment that can affect the consequence of the learning. Reflection is what permits clinicians to link medical knowledge with patient story to generate a thoughtful outcomes.^19^-^21^ This study considered the learning of basic surgical skills in novices only which is a limitation. More complex tasks and effect of structured reflection on experts should also be assessed.

## CONCLUSION

Thoughtful practice includes the implicit individual understanding. Combining the skill lab training with structured reflection can help to acquire that skill more accurately. Students who practised structured reflection made less mistakes in consecutive performances.

### Authors’ Contribution:

**TF** conceived and did statistical analysis & writing of manuscript.

**FA and TF** did the data collection

**RAH, UM,** designed and edited the manuscript.

**TF** takes the responsibility and is accountable for all aspects of the work in ensuring that questions related to the accuracy or integrity of any part of the work are appropriately investigated and resolved.
